# A systematic review of MEG‐based studies in Parkinson's disease: The motor system and beyond

**DOI:** 10.1002/hbm.24562

**Published:** 2019-03-07

**Authors:** Lennard I. Boon, Victor J. Geraedts, Arjan Hillebrand, Martijn R. Tannemaat, Maria Fiorella Contarino, Cornelis J. Stam, Henk W. Berendse

**Affiliations:** ^1^ Amsterdam UMC, location VUmc, Department of Neurology, Amsterdam Neuroscience Amsterdam the Netherlands; ^2^ Amsterdam UMC, location VUmc, Department of Clinical Neurophysiology and Magnetoencephalography Center, Amsterdam Neuroscience Amsterdam the Netherlands; ^3^ Department of Neurology Leiden University Medical Center Leiden the Netherlands; ^4^ Department of Neurology Haga Teaching Hospital The Hague The Netherlands

**Keywords:** magnetoencephalography, motor network, network analysis, Parkinson's disease, whole‐brain

## Abstract

Parkinson's disease (PD) is accompanied by functional changes throughout the brain, including changes in the electromagnetic activity recorded with magnetoencephalography (MEG). An integrated overview of these changes, its relationship with clinical symptoms, and the influence of treatment is currently missing. Therefore, we systematically reviewed the MEG studies that have examined oscillatory activity and functional connectivity in the PD‐affected brain. The available articles could be separated into motor network‐focused and whole‐brain focused studies. Motor network studies revealed PD‐related changes in beta band (13–30 Hz) neurophysiological activity within and between several of its components, although it remains elusive to what extent these changes underlie clinical motor symptoms. In whole‐brain studies PD‐related oscillatory slowing and decrease in functional connectivity correlated with cognitive decline and less strongly with other markers of disease progression. Both approaches offer a different perspective on PD‐specific disease mechanisms and could therefore complement each other. Combining the merits of both approaches will improve the setup and interpretation of future studies, which is essential for a better understanding of the disease process itself and the pathophysiological mechanisms underlying specific PD symptoms, as well as for the potential to use MEG in clinical care.

## 1 INTRODUCTION

Parkinson's disease (PD) is the second most common neurodegenerative disease after Alzheimer's disease, with a global disease burden of more than five million people (Olanow, Stern, & Sethi, 2009). The neuropathological hallmark of PD is the deposition of Lewy bodies, of which alpha synuclein is the main constituent. Nigrostriatal dopaminergic neurons are notoriously affected, and loss of these neurons leads to prominent motor features that can be treated symptomatically using levodopa suppletion and deep brain stimulation (DBS). In early disease stages, the alpha synuclein depositions mainly affect the brainstem and the surviving neurons of the nigrostriatal dopamine system, and extend to widespread cortical brain regions in later disease stages (Braak et al., 2003). PD is therefore increasingly recognized as a whole‐brain disease with functional disturbances at both subcortical and cortical levels, and is characterized clinically by both motor and nonmotor symptoms.

The past two decades have seen rapid developments in functional imaging techniques aimed at the detection, characterization and localisation of brain activity. These techniques have yielded important insights into the neuronal mechanisms that may underlie PD and its broad range of clinical symptoms. One such technique is magnetoencephalography (MEG), which noninvasively records the weak magnetic fields that are induced by electrical activity in the cerebral cortex (Cohen, 1968, 1972) and subcortical structures (Boon, Hillebrand, Dubbelink, Stam, & Berendse, 2017; Hillebrand et al., 2016; Jha et al., 2017). MEG's high temporal resolution can be used to study neuronal activity as well as functional interactions between distinct brain regions in great detail (Baillet, 2017).

Using MEG, PD‐related neurophysiological characteristics have been studied both within the motor system and for the brain as a whole. MEG analyses aimed at motor networks are spatially restricted to the motor cortex and are usually performed in source‐space. They can be combined with neurophysiological signals of different origin, such as muscle activity recorded using electromyography (EMG; Timmermann et al., 2003; Volkmann et al., 1996) or local field potentials (LFPs) from the subthalamic nucleus (STN) recorded during DBS (Hirschmann et al., 2011; Litvak et al., 2011)). The study of whole‐brain networks using MEG generally involves resting state recordings. Roughly three different approaches have been used in the analysis of whole‐brain networks: the analysis of oscillatory brain dynamics using measures of band‐limited power or peak frequency, investigation of functional (or directed/effective (Friston, 2011)) connectivity (FC) between brain areas, and assessment of the topological organization of brain networks.

MEG studies increasingly use source reconstruction techniques, such as beamforming, to project the extracranially recorded (sensor‐level) signals to source‐space. In sensor‐level analysis, several factors that may lead to erroneous estimates of functional connectivity should be considered. Multiple sensors pick up the signal from a single source because of volume conduction (the transmission of electromagnetic fields from a primary current source through biological tissue) and field spread (multiple sensors picking up activity of a common source). In addition, the same sensor picks up signals of multiple sources due to signal mixing. Moreover, the neuronal generators are generally not located directly underneath the sensor with the maximum power (particularly for axial gradiometers). The source‐level approach can resolve some of these ambiguities and enables interpretation of the functional results in an anatomical context (Baillet, Mosher, & Leahy, 2001; Brookes et al., 2007; Hillebrand, Barnes, Bosboom, Berendse, & Stam, 2012; Hillebrand, Singh, Holliday, Furlong, & Barnes, 2005; Schoffelen & Gross, 2009).

So far, review articles tend to treat motor network‐focused studies (Burciu & Vaillancourt, 2018; Magrinelli et al., 2016) and whole‐brain studies (Cozac et al., 2016) separately. Although some efforts have been made to relate findings from motor networks to nonmotor symptoms (Oswal, Brown, & Litvak, 2013b), it is unknown to what extent findings from motor networks and whole‐brain networks can be compared and if so, which similarities and discrepancies are present. A full understanding of the neurophysiological changes associated with PD is a stepping‐stone toward the development of biomarkers and novel therapies that are urgently needed. Therefore, we set out to systematically review the MEG literature on PD not only to provide an overview of the neurophysiological characteristics of PD, their relationship with clinical symptoms, the effect of disease progression, and the influence of treatment on these characteristics, but also to explore how the results of motor network studies and whole‐brain approaches can be integrated.

## 2 METHODS

We performed this systematic review of the MEG literature in PD in accordance with the Preferred Reporting Items for Systematic Reviews and Meta‐Analyses (PRISMA) guidelines (Liberati et al., 2009). We carried out web‐based searches using medical databases: PubMed, Embase, Web of Science, Emcare, Academic Search Premier, and ScienceDirect. We used combinations of the key‐words MEG and PD. The full search strategies can be found in Supplement A. References up to October 15, 2018 (date of latest search) were used for further study. Two researchers (LIB and VJG) independently screened all articles on title and abstract using the following inclusion criteria: original research article, published in English or Dutch, including a separate cohort of a minimum of five PD patients, and quantification of at least one MEG‐parameter.

Although the underlying sources of MEG and EEG are the same, these techniques measure different components of the generated electromagnetic fields (resulting in different sensitivity profiles (Goldenholz et al., 2009)). In addition, MEG is more suitable for source‐space analysis than EEG (Baillet, 2017), as it typically uses a higher number of sensors and is less affected by the details of the volume conductor. Even though neurophysiological information obtained using both techniques might be complimentary, a direct comparison would be challenging. We have therefore chosen to limit this review to MEG studies in PD (see Geraedts et al. (2018) for a recent review of quantitative electroencephalography (EEG) studies in PD (Geraedts et al., 2018)). Studies in which data analysis was confined to evoked fields were excluded, but studies aimed at induced/event‐related MEG activity were included. Induced/event‐related activity differs from evoked fields by not being phase‐locked to a certain stimulus (David, Kilner, & Friston, 2006). Cohen's kappa for inter‐rater agreement was calculated during this selection process. In case of disagreement, relevant sections were reread until agreement was reached.

Next, both reviewers evaluated the full‐text of all included articles using the Joanna Briggs Institute (JBI) checklist for case series, extended with an item addressing clear reporting of MEG data acquisition and analysis (see Supporting Information). Articles had to score a minimum of five points (indicating a sufficient quality study) to be included in this review, of which at least one point was scored on the first three items, at least two points on item 4–8, and one point on item 11. In this descriptive review, we chose to include a much‐cited article (Timmermann et al., 2003) that did not fulfill the latter (item 11) more stringent criteria on conducting and reporting the MEG research. Nonetheless, the importance of clear reporting of MEG data acquisition and analysis procedures is obvious (Gross et al., 2013). We subdivided the included articles into two main groups according to the brain network the analysis was focused on: motor network‐focused, in which we treated the tremor network as a sub‐category, and whole‐brain network focused. Since a series of articles on the neurophysiological basis of neuronal entrainment in PD (Te Woerd, Oostenveld, Bloem, De Lange, & Praamstra, 2015; Te Woerd, Oostenveld, De Lange, & Praamstra, 2014; Te Woerd, Oostenveld, De Lange, & Praamstra, 2017; Te Woerd, Oostenveld, de Lange, & Praamstra, 2018), as well as four other articles (Anninos, Adamopoulos, Kotini, & Tsagas, 2016; Boesveldt, Stam, Knol, Verbunt, & Berendse, 2009; Gomez et al., 2011; Suntrup et al., 2013) tended to stand alone from the rest of this review, these will not be discussed in the results section, but the main findings are provided in Table 1.

**Table 1:** Profiles of the motor‐network studies included in this review

Authors	Year	Center	*N*=	Type of PD cohort	Disease duration/stage^a^	JBI	Neurophysiological measures^b^	Source‐/sensor‐space	Main findings
Abbasi et al.	2018	Heinrich‐Heine University, Düsseldorf, Germany	17	All DBS	4–19 years	8	Spectral analysis	Source	Unilateral DBS (both 130 Hz and 340 Hz) leads to a lowering of alpha and beta power over both sensorimotor cortices. Recordings took place the day after surgery with eyes closed. No correlation with motor improvement was found.
Airaksinen et al.	2015	Helsinki University, Finland	19	All DBS	12 (5) years	6	Coherence: CMC	Sensor	STN‐DBS modified the CMC with large inter individual variability, correlation with motor improvement was inconsistent.
Hall et al.	2014	Aston University, Birmingham, UK	9	Early, DRT‐ naive	Unknown	6	Spectral analysis	Source	Contralateral M1 showed greater resting‐state beta power than ipsilateral M1 in PD. zolpidem normalized the ratio between left and right. Normalization correlated positively with improvement in UPDRS‐III scores. M1 beta power differences during different phases of movement (a.o. PMBR), normalized after zolpidem.
Heinrichs‐Graham et al.	2014a	University of Nebraska, USA	15		1–9 years	7	Spectral analysis FC: PLV	Source	PD (DRT OFF) vs controls: ‐power: Significantly lower beta band power in bilateral motor regions. After DRT, this largely normalizes. ‐FC: Increased synchronicity between motor cortices, partially normalized by DRT.
Heinrichs‐Graham et al.	2014b	University of Nebraska, USA	13		1–9 years	6	ERD, PMBR power	Source	Controls: Alpha and beta band desynchronization prior to and during movement. PD patients: Significantly lower response amplitudes. Trend toward lower amplitude PMBR
Heinrichs‐Graham et al.	2017	University of Nebraska, USA	23		0–16 years (mean 6.5)	6	ERD, PMBR power	Source	Response amplitudes were affected more severely in PD patients suffering from right‐dominant disease
Hirschmann et al.	2011	Heinrich‐Heine University Düsseldorf, Germany	8	All DBS	11–26 years	6	Coherence: Cortex‐STN	Source	Cortical sources coherent with oscillations STN in PD DBS patients: ‐Alpha band: Ipsilateral temporal regions ‐Beta band: Ipsilateral sensorimotor and adjacent premotor cortex
Hirschmann et al.	2013a	Heinrich‐Heine University Düsseldorf, Germany	11	All DBS	7.7 (3.4) years	6	‐Spectral analysis ‐CMC and cortico‐cortical coherence	Both	Tremor‐associated increase in STN‐M1 coherence correlated positively with tremor severity. Beta band power in cortical motor regions lower during tremor. CMC was unaffected by DRT.
Hirschmann et al.	2013b	Heinrich‐Heine University Düsseldorf, Germany	10	All DBS	15.5 (5.2) years	6	Cortex‐STN coherence and CMC	Source	Beta band motor cortex‐STN coherence reduced by DRT, but no change upon movement contralateral limb. Alpha and beta band CMC reduced during repetitive movement compared to static contraction forearm, not affected by DRT. STN‐cortical and beta band CMC negatively correlated with akinesia/rigidity during dopamine OFF state.
Jha et al.	2017	UCL London, UK	7	All DBS	9–25 years	7	Coherence: Cortex‐PPN	Source	Alpha band coherence between the PPN and posterior brain stem and cingulum. Beta band coherence between PPN and medial frontal wall, SMA and primary motor cortex
Krause et al.	2013	Heinrich‐Heine University Düsseldorf, Germany	10	Early	1.9 (0.5) years	8	CMC	Source	tACS of the motor cortex at beta frequency (20 Hz), but not at 10 Hz, attenuated beta band CMC during isometric contraction and reduced performance (amplitude variability) of a finger tapping task in PD, but not in controls.
Litvak et al.	2011	UCL London, UK	17	All DBS	8–17 years	6	Coherence: Cortex‐STN (incl. Directed coherence)	Source	Cortical sources coherent with oscillations STN in PD DBS patients: ‐Alpha band: Ipsilateral temporo‐parietal regions. ‐Beta band: Ipsilateral anterior parietal and frontal cortex. ‐STN activity predominantly led by cortical activity in both frequency bands. ‐No changes upon DRT
Litvak et al.	2012	UCL London, UK	13	All DBS	8–17 years	7	‐Spectral analysis ‐Coherence: Cortex‐STN ‐Granger causality	Source	Gamma‐band coherence between STN and M1, with the STN mostly driving the cortex. Upon movement of the hand, gamma band STN‐M1 event‐related coherence increased. DRT increased gamma band coherence between the STN and M1, which correlated with the degree of improvement in bradykinesia‐rigidity.
Luoma et al.	2018	Helsinki university hospital, Finland	16	All DBS	11.9 (5.0) years	7	‐Spectral analysis ‐CMC	Sensor	‐Lowering of alpha and beta band power during DBS ON, only during resting state when the eyes were open. During eyes‐closed or a motor task: No significant difference between ON and OFF stimulation. ‐Maximum CMC over sensorimotor area contralateral to extended hand.
Mäkelä et al.	1993	Helsinki University, Finland	5		1.5–6.3 years	6	Spectral analysis	Sensor	Beta band power in cortical motor regions lower during tremor.
Meissner et al.	2018	Heinrich‐Heine University, Düsseldorf, Germany	20		5.5 (3) years	5	ERD, ERS and PMBR power	Source	PD patients performed worse than controls on motor task (motor sequence acquisition). During random presentation of the task no differences in beta band power. After learning a sequence: Less training‐related beta power suppression in motor cortex in PD versus HC. In addition, less training related theta activity in cortical motor regions, paralleling susceptibility to inference.
Oswal et al.	2013	University of Oxford, UK	17	All DBS	8–17 years	7	Coherence: STN‐cortex	Source	Alpha band coherence between temporal cortical areas and the STN reduced following movement onset: Degree of suppression in is significantly greater ON DRT than OFF DRT.
Oswal et al.	2016	University of Oxford, UK	15	All DBS	6–22 years	6	Coherence: STN‐cortex; Granger causality variant	Source	DBS relatively selectively suppressed lower beta band synchronization of activity between STN and mesial premotor regions, including SMA. Motor cortical regions “driving” STN in beta band, with different delays for lower and higher beta band.
Pollok et al.	2009	Heinrich‐Heine University Düsseldorf, Germany	10		10.9 (2.4) years Range: 4–30 years	6	CMC and cortico‐cortical coherence	Source	Oscillatory network associated with tremor comprising: Contralateral S1/M1, SMA, PMC, thalamus, S2, PPC and ipsilateral cerebellum oscillating at 8–10 Hz. M1/S1 CMC at double the tremor frequency, CMC decreased following DRT. When controls imitated a tremor, oscillatory network comparable to PD‐tremor network when observed in DRT OFF.
Pollok et al.	2012	Heinrich‐Heine University Düsseldorf, Germnany	20	Early PD, of which 10 patients DRT naive	HY stage (all): I‐II DRT naïve: 0.4–2.5 years Treated group: 1–3.5 years	8	‐Spectral analysis ‐Coherence: Cortico‐cortical	Source	In early PD: ‐Increased resting‐state S1/M1 beta band power ‐CMC did not differ between PD and controls.
Pollok et al.	2013	Heinrich‐Heine University Düsseldorf, Germany	7		11.9 (0.6)years	6	Cortico‐cortical coherence	Source	During rest in DRT ON (but not during DRT OFF): Positive correlation between disease duration and SMA–M1 coherence. During isometric contraction in DRT OFF (but not during DRT ON): Inverse correlation between UPDRS III and SMA–M1 coherence.
Salenius et al.	2002	Helsinki University, Finland	8		HY stage: I‐III	6	‐Spectral analysis ‐CMC	Source	Trend toward lower beta band power in motor cortex PD patients. Beta and gamma band CMCs during steady‐state contraction of the forearm significantly lower in PD than in controls
Te Woerd et al.	2014	Radboud University medical Centre Nijmegen, the Netherlands	12		1–12 years (mean 6)	6	ERD, ERS, PMBR power	Source	Pre‐stimulus beta band power: Patients showed a lower proportion of beta band ERD in the sensorimotor cortex *preceding* the reaction stimuli, while the proportion of beta band ERD *following* the event was larger, hence there was a change in timing of the ERD: a shift from predictive to more reactive modulation of beta band oscillations.
Te Woerd et al.	2015	Radboud University medical Centre Nijmegen, the Netherlands	15		7 (4) years	6	ERD, ERS, PMBR power	Source	Rhythmic auditory stimulation regime: Entrainment of slow oscillations and increases in modulation depth of beta oscillatory activity, in PD and controls. Due to increased beta ERS postmovement, that improves the predictive movement related beta suppression, reflecting a predictive mode of cue utilization.
Te Woerd et al.	2017	Radboud University medical Centre Nijmegen, the Netherlands	14		8 (5) years	6	ERD, ERS, PMBR power	Source	PD patients have demonstrated comparable auditory entrainment as controls. Therefore: Deficient entrainment in PD patients concerns the motor circuits only.
Te Woerd et al.	2018	Radboud University medical Centre Nijmegen, the Netherlands	12		7 (5) years	6	ERD, ERS, PMBR power	Sensor	PD patients showed reduced motor entrainment compared to controls during tasks containing rhythmic stimuli, even in situations encouraging entrainment. This is also reflected by beta oscillatory power changes, both regarding phase and modulation depth.
Timmermann et al.	2003	Heinrich‐Heine University Düsseldorf, Germany	6		1–21 years (mean 7)	6	CMC and cortico‐cortical coherence	Source	Tremor‐related oscillatory network, consisting of a cerebello‐diencephalic‐cortical loop and cortical motor (M1, SMA/CMA, PM) and sensory (SII, PPC) areas contralateral to the tremor hand.
van Wijk et al.	2016	UCL London, UK	33	Subset of patients previously described by Litvak et al. (2011); Litvak et al. (2012); Oswal, Brown, and Litvak (2013a)	12 (5–25) years	8	Coherence: STN‐cortex	Source	Beta band power and phase‐amplitude coupling within the STN correlated positively with severity of motor impairment (lower beta). Coherence between STN and motor cortex dominant in the high‐beta range.
Vardy et al.	2011	VUmc, Amsterdam, the Netherlands	11		5.1 (3.3) years HY stage 1.5‐III	7	Spectral analysis	Source	Cortical motor slowing during rest in correlation with cognitive UPDRS scores, whereas slowing during movement correlated best with the motor UPDRS scores.
Volkmann et al.	1996	New York University medical center, USA	7		7.8 (2.5) years HY stage I‐III	6	Coherence: CMC	Source	Tremor network contralateral to the 3–6 Hz Parkinson resting tremor, involving the diencephalic level (assumed to be the thalamus), lateral PMC, S1 and M1

a Mean (standard deviation) or range (..‐..)

b Neurophysiological measures relevant for this review; explanation of the measures can be found in Table 3.

*Note*. CMC: cortico‐muscular coherence; DBS: deep brain stimulation; DRT: dopamine replacement therapy; ERD: event‐related desynchronization; ERS: event‐related synchronization; FC: functional connectivity; HY stage: Hoehn and Yahr stage; JBI: Joanna Briggs Institute (score); N: number of PD subjects studied; PD: Parkinson's disease; PLV: phase locking value; PMC: premotor cortex; PMBR: postmovement beta rebound; PPC: posterior parietal cortex; PPN: pedunculopontine nucleus; S1/M1; primary sensorymotor cortex; SMA: supplementary motor area; STN: subthalamic nucleus; tACS: transcranial alternating current stimulation; UPDRS: Unified Parkinson's disease Rating Scale.

## 3 RESULTS

### 3.1 Search results and study characteristics

**Figure 1:**
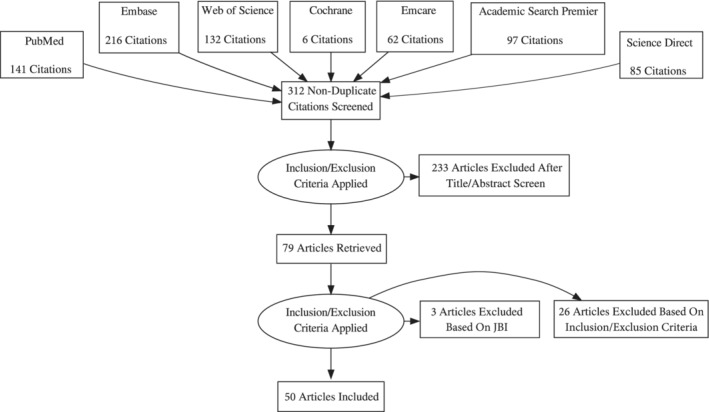
Flowchart for inclusion of studies

Figure 1 shows the selection procedure with corresponding numbers of publications. 312 articles matched the search terms and were included for title and abstract screening, leading to 79 articles meeting the pre‐specified in‐ and exclusion‐criteria (Kappa = 0.832). These articles were selected for full‐text analysis, risk of bias assessment was performed, and data extraction took place. Three articles were excluded based on the JBI checklist (see Supporting Information) and 26 articles were excluded based on the inclusion/exclusion criteria. Eventually, 50 articles were included for review. Frequency bands were defined as follows: delta (0.5–4 Hz), theta (4–8 Hz), alpha1 (8–10 Hz), alpha2 (10–13 Hz), beta (13–30 Hz) and gamma (30–48 Hz). In several motor‐network focused studies, the beta band has been divided into low and high‐beta. The upper limit of the low‐beta band is 20 Hz (Hirschmann et al., 2011; van Wijk et al., 2016), 21 Hz (Oswal et al., 2016), 22 Hz (Abbasi et al., 2018), or 25 Hz (Airaksinen et al., 2015). An explanation of the neurophysiological measures described in the reviewed articles is presented in Table 3.

### 3.2 Motor network‐focused research

**Figure 2:**
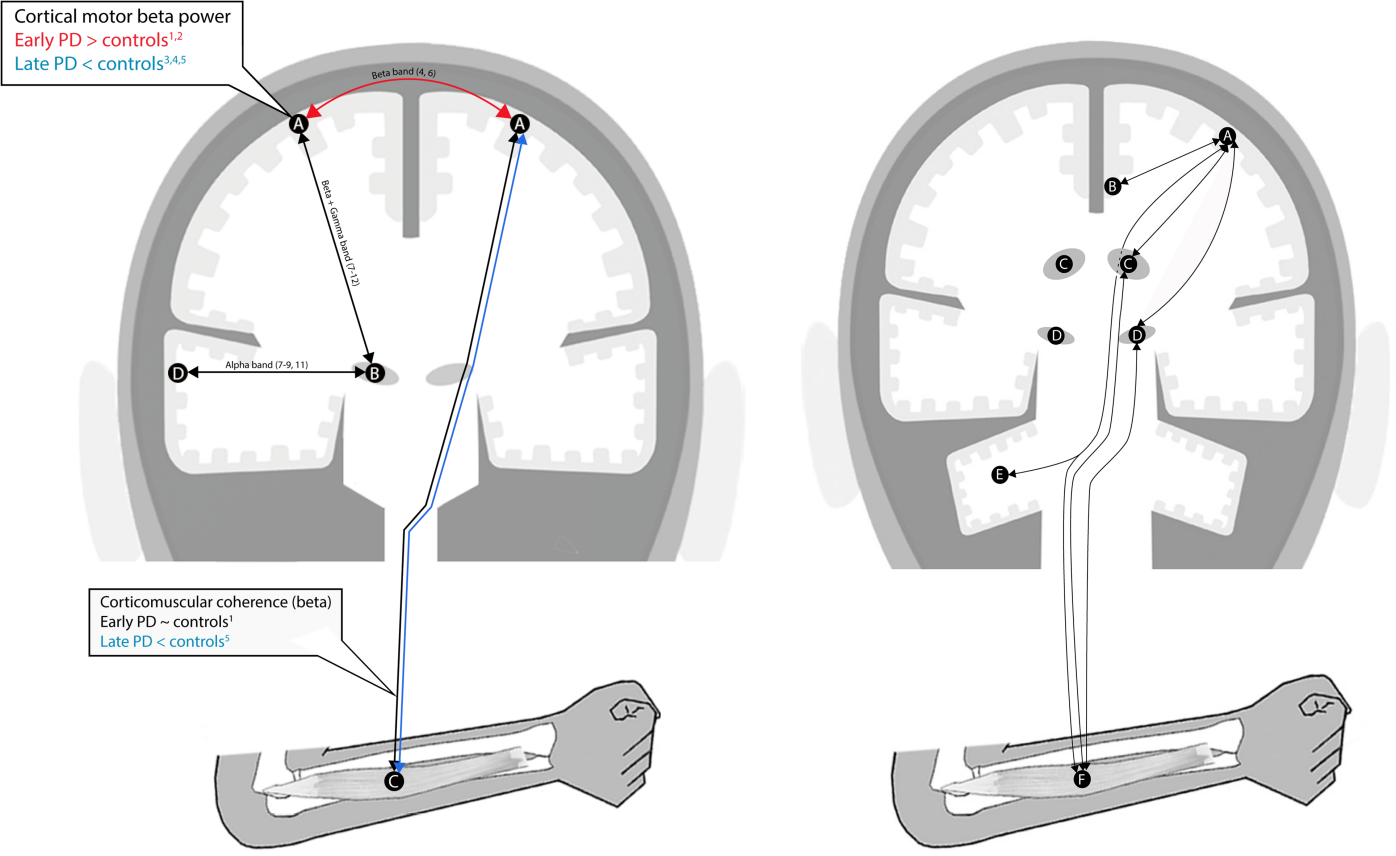
(in color) Overview of main findings in motor network‐focused research. A schematic representation of a coronal view of the brain, combined with the forearm muscle extensor digitorum communis. All displayed findings involve undirected functional connectivity, depicted using lines with double arrow heads. A: motor cortex; B: subthalamic nucleus; C: forearm muscle; D: temporal cortex. *Red* and *blue* represent higher respectively lower values found in PD patients compared with controls; *Black* lines represent no significant difference between PD patients and controls, or no comparison with a control group. References: (Hall et al., 2014; Heinrichs‐Graham, Kurz, et al., 2014; Hirschmann et al., 2011; Hirschmann, Ozkurt, et al., 2013; Litvak et al., 2011; Litvak et al., 2012; Oswal, Beudel, et al., 2016; Pollok et al., 2012; Pollok et al., 2013; Salenius et al., 2002; van Wijk et al., 2016; Vardy et al., 2011). (b)Overview of main findings in tremor network‐focused research. A schematic representation of a coronal view of the brain, combined with the forearm muscle extensor digitorum communis. All displayed findings involve coherence at tremor frequency and its (sub)harmonics. A: sensorimotor and premotor cortex; B: cingulate motor area; C: thalamus; D: subthalamic Nucleus; E: cerebellum; F: forearm muscle. Not depicted in this figure: Posterior parietal cortex. References: (Hirschmann, Hartmann, et al., 2013; Pollok et al., 2009; Timmermann et al., 2003; Volkmann et al., 1996)

A summary of the data extraction and risk of bias assessment of the motor network‐focused articles can be found in Table 1 and a schematic overview to place the main findings in an anatomical context are provided in Figure 2. Unless stated otherwise, motor network‐focused studies in this review have been performed in source‐space.

#### 3.2.1 Early disease stages

Larger sensori‐motor cortical (S1/M1) beta band power has been reported both in early‐stage PD patients on dopamine replacement therapy (DRT) and in medication naïve patients as compared to controls, recorded during the resting state (Pollok et al., 2012). In this study, during isometric contraction of the contralateral forearm, beta band power was suppressed in controls, but not in PD patients. Only during isometric contraction, contralateral beta band power correlated with Unified Parkinson's Disease Rating Scale (UPDRS)‐III scores in PD patients (Pollok et al., 2012). Hall and coworkers found larger resting‐state beta band power in the motor cortex contralateral to the most affected hemibody in DRT‐naïve patients. The benzodiazepine zolpidem, known for its modulating effects on PD motor symptoms, normalized the ratio in resting‐state beta band power between the “affected” and “nonaffected” motor cortex and this correlated positively with improvement in UPDRS‐III scores (Hall et al., 2014). Cortico‐muscular coherence (CMC) has been studied by correlating M1 activity with EMG signals recorded in the forearm. CMC was not different between PD patients and controls during steady‐state contraction of the forearm (Pollok et al., 2012).

#### 3.2.2 Later disease stages

Studies in later‐stage PD patients found that beta band power in cortical motor regions was lower during the resting state compared to controls (both OFF and ON DRT; Heinrichs‐Graham et al., 2014; Vardy et al., 2011). Vardy and colleagues demonstrated that slowing of event‐related beta band oscillations in the motor cortex correlated positively with UPDRS‐III scores when recorded during a motor task and with cognitive UPDRS components when recorded during the resting state (Vardy et al., 2011). DRT significantly increased cortical motor beta band power, thus having a normalizing effect (Heinrichs‐Graham, Kurz, et al., 2014). In contrast, STN‐DBS lowered alpha and low‐beta band power in the sensorimotor cortex in two studies (both a sensor‐space and a source‐space study) during eyes‐open, resting‐state (Abbasi et al., 2018; Luoma et al., 2018). However, no correlation with motor improvement has been observed. In addition, during a motor task, as well as during eyes‐closed, no differences between ON and OFF stimulation were found.

Even in the absence of stimulation, MEG data are contaminated by high‐amplitude, low frequency artifacts mainly originating from the influence of cardiovascular pulsations and breathing on the percutaneous extension wire (before implantation of a stimulator; Litvak et al., 2010), and the stimulator itself (Oswal et al., 2016). Upon stimulation, electromagnetic artifacts generated by the stimulator, such as jump artifacts and ringing artifacts, obscure neuronal activity (see (Oswal, Jha, et al., 2016) for a detailed description of DBS‐artifacts). However, MEG recordings are still technically feasible as DBS artifacts can be minimized using spatial filters (Airaksinen et al., 2011; Cao et al., 2015; Cao et al., 2017), beamforming techniques (Mohseni et al., 2010; Oswal, Jha, et al., 2016), or independent component analyses in combination with mutual information (Abbasi, Hirschmann, Schmitz, Schnitzler, & Butz, 2016). For a recent review on the effect of DBS on multiple diseases, studied using MEG, see (Harmsen, Rowland, Wennberg, & Lozano, 2018).

When studying induced MEG activity, prior to movement onset, in healthy individuals a desynchronization in cortical motor oscillations (beta band) occurs, that disappears during the actual execution of the movement: Event‐related desynchronization (ERD). This is followed by a postmovement beta band rebound: Event‐related synchronization (ERS; Gaetz, Macdonald, Cheyne, & Snead, 2010; Jurkiewicz, Gaetz, Bostan, & Cheyne, 2006). In PD patients OFF DRT, ERD, and ERS response amplitudes are reportedly lower compared to controls (Heinrichs‐Graham et al., 2014), mainly for right‐dominant diseased patients (Heinrichs‐Graham, Santamaria, Gendelman, & Wilson, 2017), but ON DRT these differences could not be substantiated (Meissner, Krause, Sudmeyer, Hartmann, & Pollok, 2018).

One study demonstrated higher resting‐state beta band coherence between bilateral primary cortical motor regions in PD patients compared to controls, which normalized after DRT administration (Heinrichs‐Graham, Kurz, et al., 2014). In akinesia‐dominant PD patients, coherence between the ipsilateral supplementary motor area (SMA) and M1 correlated with disease duration, not with UPDRS III scores, during rest (only ON DRT). During isometric contraction of the forearm, coherence between SMA and M1 was inversely correlated with UPDRS III scores (only OFF DRT; Pollok et al., 2013).

Forearm CMCs in the beta and gamma band were demonstrated to be significantly lower in PD patients than in controls when recorded during steady‐state contraction (Salenius, Avikainen, Kaakkola, Hari, & Brown, 2002) and this correlated with higher akinesia and rigidity sub‐scores (Hirschmann et al., 2013). This difference normalized after DRT in one study (Salenius et al., 2002), but not in another (Hirschmann, Ozkurt, et al., 2013). It was speculated by Hirschmann and colleagues that this differential response to DRT was caused by the fact that tremor‐dominant PD patients were not excluded from the study from Salenius and colleagues (Hirschmann, Ozkurt, et al., 2013; Salenius et al., 2002), as CMC increases might be a characteristic of tremor alleviation (Park et al., 2009). Transcranial alternating current stimulation (tACS) of the motor cortex at beta frequency (20 Hz), but not at 10 Hz, further attenuated both the beta band CMC during isometric contraction and reduced performance (amplitude variability) on a finger tapping task in PD patients, but not in controls (Krause et al., 2013). In a sensor‐ space study on the effect of DBS on motor CMC, results varied and the correlation with improvement in motor function inconsistent (Airaksinen et al., 2015).

By combining LFP recordings with MEG recordings in STN‐DBS patients, a frequency‐dependent coherence has been demonstrated between signals from the STN and the ipsilateral S1/M1 cortex in the beta and gamma band during the resting state (Hirschmann et al., 2011; Hirschmann, Ozkurt, et al., 2013; Litvak et al., 2011; Litvak et al., 2012; Oswal, Beudel, et al., 2016; van Wijk et al., 2016). Beta coherence was most dominant in the high beta band (van Wijk et al., 2016), which was mainly located in the mesial premotor regions (Hirschmann et al., 2011; Litvak et al., 2011; Oswal, Beudel, et al., 2016). Resting‐state M1‐STN beta band coherence was inversely correlated (Hirschmann, Ozkurt, et al., 2013) or not correlated with bradykinesia/rigidity UPDRS‐III scores (DRT ON and OFF; Litvak et al., 2011. DRT increased beta band coherence between the STN and a small region in the prefrontal cortex in one study (Litvak et al., 2011), but in other studies DRT suppressed (Hirschmann, Ozkurt, et al., 2013) or did not modulate (van Wijk et al., 2016) beta band coherence between the motor cortex and the STN. In one study, stimulation of the STN suppressed resting‐state high‐beta band coupling of the STN with mesial cortical motor regions, yet the degree of suppression did not correlate with motor improvement (Oswal, Beudel, et al., 2016).

Resting‐state alpha band coherence has been observed between the STN and ipsilateral temporal cortex (Hirschmann et al., 2011; Hirschmann, Ozkurt, et al., 2013; Litvak et al., 2011; Oswal, Beudel, et al., 2016). The alpha band coherence was not influenced by arm movements in one study (Hirschmann, Ozkurt, et al., 2013), but decreased upon movement in another study (in DRT ON more than in DRT OFF; Oswal et al., 2013a). DRT and DBS did not influence the resting‐state alpha band coupling (Hirschmann et al., 2011; Litvak et al., 2011; Oswal, Beudel, et al., 2016). The former authors suggested that the identified alpha band network may reflect nonmotor functioning, for example auditory processing involving the (8–10 Hz) tau rhythm in the auditory cortex (Weisz, Hartmann, Müller, Lorenz, & Obleser, 2011), or attentional processes (Hirschmann et al., 2011; Litvak et al., 2011; Oswal et al., 2013b).

#### 3.2.3 Tremor network‐focused research

Tremor most likely involves neuronal mechanisms different from those underlying bradykinesia and rigidity, as the latter symptoms worsen at the same rate as gait and balance impairments, whereas tremor does not (Louis et al., 1999). MEG studies aimed at revealing PD‐related tremor networks have identified a number of brain regions with oscillatory activity that is coherent with forearm EMG signals at tremor frequency. First, a motor network contralateral to the 3–6 Hz Parkinson resting tremor has been identified involving the diencephalic level (likely corresponding to the thalamus), the lateral premotor cortex, S1 and M1 (Volkmann et al., 1996). Thereafter, cortico‐cortical coherence analysis with contralateral M1 as a seed region (i.e., in which signals from the selected brain region are used to calculate correlations with the rest of the brain) revealed harmonic involvement (at single and double frequency) of the ipsilateral cerebellum, contralateral cingulate motor area (CMA) and contralateral posterior parietal cortex (PPC; Pollok et al., 2009; Timmermann et al., 2003). Over the years, several interesting additional observations have been made: (a) using MEG in combination with LFP recordings in DBS‐patients, a muscular‐STN‐M1 coupling was found during tremor (Hirschmann et al., 2013). (b) when controls were asked to imitate a tremor, an oscillatory network could be identified that is comparable to the PD‐tremor network observed in the dopamine‐OFF state (Pollok et al., 2009). (c) beta band power in cortical motor regions was lower during simultaneous measurement of an intermittent tremor (Hirschmann, Hartmann, et al., 2013; Makela, Hari, Karhu, Salmelin, & Teravainen, 1993).

### 3.3 Whole‐brain focused research

**Figure 3:**
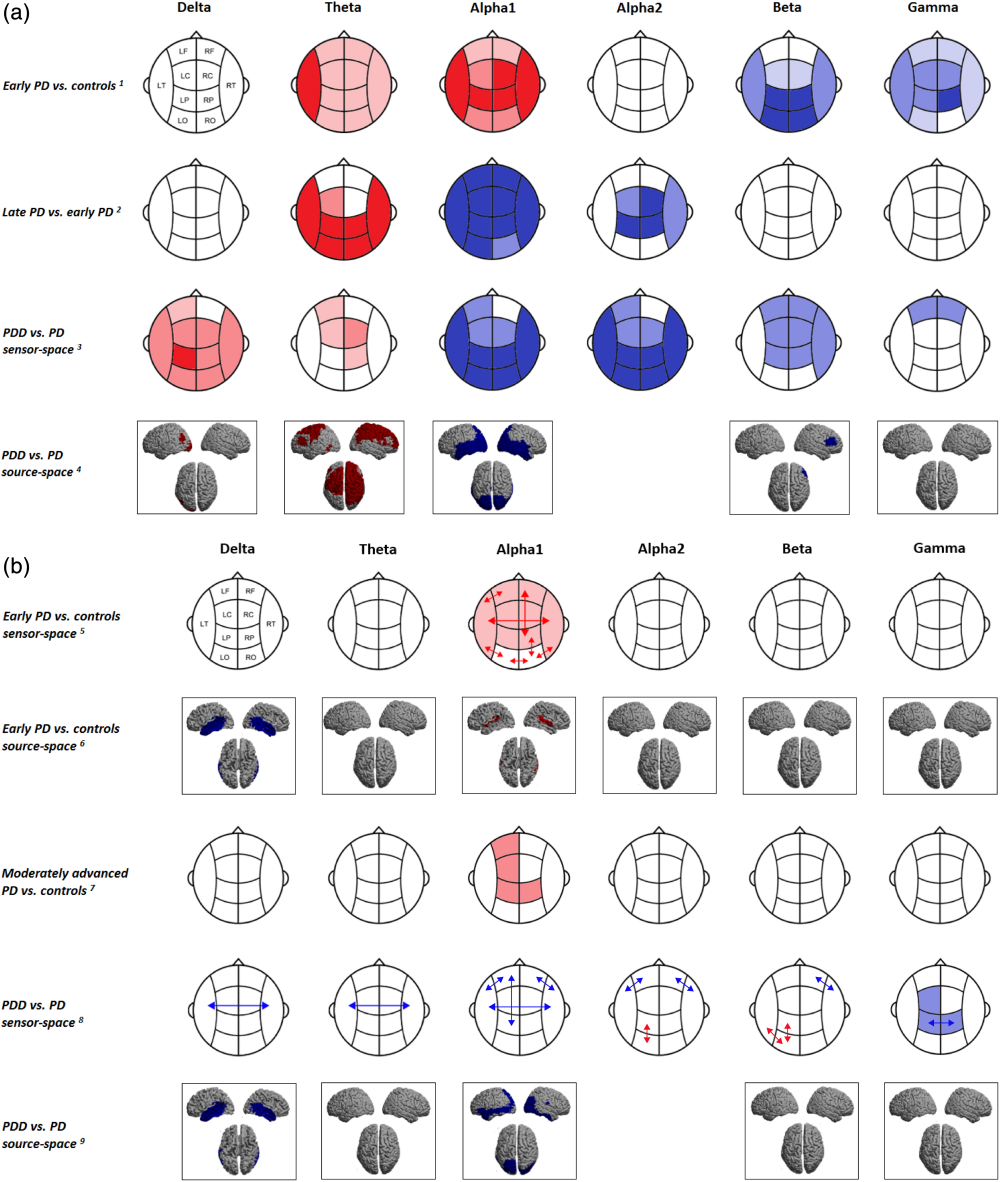
(in color) Overview of main findings in whole brain network‐focused research: Band power. Schematic representation of observed statistical differences in relative band power between groups. Both sensor‐space and source‐space analyses are included in the figure. In case of sensor‐space analysis, the brain region underlying the relevant sensor was colored. In case of source‐space analysis results for each ROI are displayed as a color‐coded map on a parcellated template brain viewed from, in clockwise order, the left, right, and top. An area is colored red when the mean power early PD > controls, late PD > early PD, and PDD > PD and blue when the difference was in the opposite direction. The three color codes of magnitudes (from light to dark) illustrate the effect size of the observed difference. Areas that did not show statistically significant differences are represented in white/gray. In the study by (Ponsen et al., 2012) the alpha1 and alpha2 band were combined. PD, Parkinson's disease without dementia; PDD, Parkinson's disease related dementia; L or R, cortical area on the left (L) or right (R) side of the head; C, central; F, frontal; O, occipital; P, parietal; T, temporal. Figure adapted from (Bosboom et al., 2006; Olde Dubbelink et al., 2013a; Ponsen et al., 2012; Stoffers et al., 2007). (b) (in color) Overview of main findings in whole brain network‐focused research: Functional connectivity. Schematic representation of observed statistical differences. In case of a sensor‐space analysis differences are depicted for local (colored regions) and interregional (arrows) functional connectivity (FC; synchronization likelihood and phase lag index) between groups. In case of a source‐space analysis differences in FC from one ROI to the rest of the brain (using phase lag index) are displayed as a color‐coded map on a parcellated template brain viewed from, in clockwise order, the left, right, and top. An area is colored red when the FC of early PD > controls, moderately advanced PD > controls, and PDD > PD and blue when the difference was in the opposite direction. Areas that did not show statistically significant differences are represented in white/gray. In the study by Ponsen et al. (2012) the alpha1 and alpha2 band were combined. PD, Parkinson's disease without dementia; PDD, Parkinson's disease related dementia; L or R, cortical area on the left (L) or right (R) side of the head; C, central; F, frontal; O, occipital; P, parietal; T, temporal. Figure adapted from (Bosboom, Stoffers, Wolters, et al., 2009; Cao et al., 2018; Olde Dubbelink et al., 2013b; Ponsen et al., 2012; Stoffers, Bosboom, Deijen, et al., 2008)

A summary of the data extraction and risk of bias assessment of the whole‐brain focused articles can be found in Table 2 and a schematic overview of the main findings is provided in Figure 3. Unless stated otherwise, the whole‐brain focused studies have been performed in sensor‐space.

**Table 2:** Profiles of the whole‐brain studies included in this review

Authors	Year	Center	*N*=	Type of PD cohort	Disease duration/stage^a^	JBI	Neurophysiological measures^b^	Source/sensor space	Main findings
Airaksinen et al.	2012	Helsinki University, Finland	11	All DBS	7–19 years	6	Spectral analysis	Sensor	STN‐DBS modulated alpha (occipital) and beta band (central sulcus) power. Lowering of the latter correlated positively with relief of rigidity.
Anninos et al.	2016	Democritus University of Thrace, Alexandropouli, Greece	10	All male	Unknown	7	Spectral analysis	Sensor	TMS over the five main cortical brain regions led to nonsignificant increases in PD‐related abnormally low peak frequency.
Boesveldt et al.	2009	VUmc, Amsterdam, the Netherlands	20		HY stage I‐III	8	‐spectral analysis ‐FC: SL	Sensor	Upon odor stimulation task: ‐PD‐related decrease in alpha power. ‐controls: Decrease in local beta band SL. PD: Decrease in intrahemispheric alpha2 band SL.
Boon et al.	2017	VUmc, Amsterdam, the Netherlands	34	6 PDD	HY stage II‐III 11.9 (3.8) years	7	FC: dPTE	Source	Lower resting‐state beta band directed connectivity (dPTE) in posterior brain regions in PD. lower posterior dPTE values correlated with poor global cognitive performance.
Bosboom et al.^c^	2006	VUmc, Amsterdam, the Netherlands	26	13 PD, 13 PDD	PD: 9.69 (4.5) years, HY stage 2.5 PDD: 11.2 (4.0) years, HY stage 2.9	7	Spectral analysis	Sensor	PD: Slowing of resting‐state brain activity involving theta, beta and gamma bands. PDD: Further slowing of resting‐state brain activity, additionally involving delta and alpha bands, as well as a lower reactivity to eye‐opening.
Bosboom et al.	2009a	VUmc, Amsterdam, the Netherlands	8	All PDD	12.8 (2.6) years HY stage II‐IV	7	Spectral analysis	Sensor	Rivastigmine administration to PDD patients: Shift spectrum toward higher frequencies: Increase in parieto‐occipital and temporal alpha power and a diffuse increase in beta power, together with a decrease in fronto‐central and parieto‐occipital delta power.
Bosboom et al.^c^	2009b	VUmc, Amsterdam, the Netherlands	26	Cohort previously described by Bosboom et al. (2006)	Previously described	7	FC: SL	Sensor	PDD vs. PD: ‐lower fronto‐temporal SL in alpha band and lower intertemporal SL in delta, theta and alpha1 band. ‐higher left sided parieto‐occipital SL in alpha2 and beta band.
Cao et al.	2015	Shanghai Jiatong University, China	32	16 PD, 16 PD‐DBS	PD: 2–30 years PD‐DBS 4–13 years	8	Spectral analysis	Sensor	PD vs controls: General occipitotemporal slowing. PD‐DBS first week after STN‐DBS placement: No band power differences upon stimulation. Long‐term STN‐DBS: Average cortical frequency increased upon stimulation. Relative 9–13 Hz power over left hemisphere correlated positively with UPDR‐III scores in DBS‐ON state.
Cao et al.	2017	Shanghai Jiatong University, China	27	13 dB	PD: 11.3 (1.3) years PD‐DBS: 9.4 (1.3) years	7	Spectral analysis	Sensor	PD vs. controls: Increase in absolute power between 8 and 30 Hz. Upon STN stimulation: Frontal/parietal increase in lower gamma band power (34–38 Hz) and higher gamma band power (55–65 Hz). Improvement of motor symptoms correlated with alpha and beta band power suppression over right temporal area.
Gomez et al.	2011	University of Valladolid, Spain	18	Early	<2 years	7	Complexity of oscillations	Sensor	PD patients have lower complexity values in MEG signals than controls: Statistical group differences for all (10) major cortical regions.
Olde Dubbelink et al.	2013a	VUmc, Amsterdam, the Netherlands	49	Longitudinal, 3 PDD (last time point)	Baseline: 5.4 (3.5) years	6	Spectral analysis	Sensor	PD patients vs. controls: ‐slowing dominant peak frequency ‐global increase in low frequency and decrease in high frequency relative spectral power over time. ‐degree of slowing associated with clinical measures of disease progression, in particular cognitive decline.
Olde Dubbelink et al.^d^	2013b	VUmc, Amsterdam, the Netherlands	43	Longitudinal 4 PDD (last time point)	Baseline: 5.2 (3.6) years	6	‐spectral analysis ‐FC: PLI	Source	PD patients vs. controls: ‐baseline: Lower delta and higher alpha1 FC in temporal regions ‐longitudinal follow‐up (4 years): Decrease alpha1 and alpha2 band FC ‐motor and cognitive dysfunction correlated positively to the latter.
Olde Dubbelink et al.^d^	2014a	VUmc, Amsterdam, the Netherlands	43	Cohort previously described by Olde Dubbelink et al. (2013b)	Previously described	6	‐graph analysis ‐minimum Spanning tree (MST)	Source	Early‐stage PD: Lower local integration delta band, preserved global efficiency. Longitudinal analysis: More random brain topology. Local integration (multiple frequency bands) and global efficiency (alpha2) affected. Worsening global cognition associated with more random topology in the theta band, motor dysfunction was associated with lower alpha2 global efficiency. MST analysis: a progressive decentralization of the network configuration, in correlation with deteriorating motor function and cognitive performance
Olde Dubbelink et al.	2014b	VUmc, Amsterdam, the Netherlands	63	Longitudinal 19 PDD (last time point)	Baseline: PD: 60.9 (6.5) PDD: 66.0 (5.2)	7	Spectral analysis	Source	Addition of neurophysiological markers to neuropsychological tests substantially improved prediction of the risk of conversion to PDD. Lower beta power was associated with the greatest risk of developing dementia.
Ponsen et al.^c^	2012	VUmc, Amsterdam, the Netherlands	26	Cohort previously described by (Bosboom et al., 2006)	Previously described	6	Spectral analysis FC: PLI	Source	PDD vs. PD: ‐lower alpha and beta band power in occipito‐parieto‐temporal and frontal regions. ‐lower FC in delta and alpha bands in respectively the fronto‐temporal and occipito‐parieto‐temporal areas. ‐FC between pairs of regions generally weaker in delta and alpha band, stronger in theta band.
Stoffers et al.^e^	2007	VUmc, Amsterdam, the Netherlands	70		HY stage I‐III 5.5 (3.7) years	7	Spectral analysis	Sensor	Widespread slowing of resting‐state brain activity in de novo, untreated PD patients.
Stoffers et al.^e^	2008a	VUmc, Amsterdam, the Netherlands	70	Cohort previously described by Stoffers et al. (2007)	Previously described	6	Spectral analysis FC: SL	Sensor	Drug‐naive PD patients vs controls: Overall increase in alpha1 SL Moderately advanced PD: Increased theta, alpha1, alpha2 and beta SL, particularly with regard to local SL. Total cohort: Disease duration positively associated with alpha2 and beta SL, and severity of motor disease with theta and beta SL measures.
Stoffers et al.	2008b	VUmc, Amsterdam, the Netherlands	37		HY stage I‐III 8.0 (2.7) years	7	Spectral analysis FC: SL	Sensor	Elevated levels of cortico‐cortical FC are increased even further by an acute DRT challenge, in parallel with motor improvement. Increases involved local FC (4–30 Hz) and intra‐ and interhemispheric FC (13–30 Hz).
Suntrup et al.	2013	University of Münster, Germany	20	10 dysfagia and 10 nondysfagia PD patients	‐Dysfagic PD: 5.3 (6.7) ‐nondysfagic PD: 8.2 (4.4)	7	Event (swallowing)‐related power	Source	A strong decrease in overall task‐related cortical activation was found in all PD patients, most prominent in dysfagic patients. In nondysfagic patients a compensatory activation toward lateral motor, premotor and parietal cortices seems to take placed upon swallowing, whereas the supplementary motor area was markedly reduced in activity.
Wiesman et al.	2016	University of Nebraska, USA	16		1–9 years HY stage 1.5‐III	6	Spectral analysis Coherence: CMC	Source	During a memory task, a significant reduction in alpha FC between left inferior frontal cortices and left supramarginal/superior temporal cortices in PD compared to controls.

a Mean (standard deviation) or range (..‐..)

b Neurophysiological measures relevant for this review; explanation of the measures can be found in Table 3

*Note*. DBS: deep brain stimulation; dPTE: directed phase transfer entropy; DRT: dopamine replacement therapy; ERD: event‐related desynchronization; PLI: phase lag index; FC: functional connectivity; PDD: Parkinson's disease dementia; HY stage: Hoehn & Yahr stage; JBI: Joanna Briggs Institute (score); MST: minimum spanning tree; N: number of PD subjects studied; PD: Parkinson's disease; PLV: phase locking value; SL: synchronization likelihood; STN: subthalamic nucleus; TMS: transcranial magnetic stimulation.

^c^, ^d^, ^e^: Articles that have studied the same patient cohort.

**Table 3:** Definitions of the neurophysiological measures described in the review

Category	Measure	Interpretation
Oscillatory behaviour	Band power	Average spectral power in a particular frequency band.
	Mean frequency	Average frequency of the spectrum within a given frequency range.
	Peak frequency	Dominant frequency in the power spectrum, within a given frequency range (e.g., 6–15 Hz in (Airaksinen et al., 2012); 4–13 Hz in (Olde Dubbelink et al., 2013a)).
Complexity	Lempel‐Ziv complexity	Related to the number of distinct patterns and the rate of their occurrence along a given sequence. A high value indicates a high variation of the binary signal (Lempel & Ziv, 1976).
Functional connectivity	Coherence	The degree of similarity of frequency components of two time series. Field spread and volume conduction, as well as power, influence the estimate. High values indicate strong functional connectivity (White & Boashash, 1990).
	Phase lag index	Instantaneous phases of two time series are compared at each time point and the asymmetry of the distribution of the phase differences between these time series is quantified. A high value indicates that there is a consistent nonzero (modulus *π*) phase relation between the two time series, indicative of functional coupling (Stam, Nolte, & Daffertshofer, 2007). Relatively insensitive to the effects of field spread and volume conduction.
	Phase locking value	Reflects the consistency of the phase covariance between two signals in a frequency range over time (phase‐locking). Field spread/volume conduction affect the estimate (Lachaux, Rodriguez, Martinerie, & Varela, 1999).
	Synchronization likelihood	The strength of synchronization between two time series based on state‐space embedding. High values indicate strong functional connectivity, but field spread/volume conduction affects the estimate (Stam & Van Dijk, 2002).
Directed functional connectivity	Directed phase transfer entropy	Based on the Wiener–Granger Causality principle, namely that a source signal has a causal influence on a target signal if knowing the past of both signals improves the ability to predict the target's future compared with knowing only the target's past: dPTE was implemented as a ratio between “incoming” and “outgoing” information flow (Hillebrand et al., 2016).
	Granger causality	Quantifies whether the past of one time series contains information that helps to predict the future of another signal. Does not capture nonlinear effects and requires construction of a model of the data (Granger, 1969).
	Partial directed coherence	Based on the notion of Granger causality. Frequency‐domain approach to describe the (direction of) relationships between time series. Decomposes the relationships into “feedforward” and “feedback” aspects (Baccala & Sameshima, 2001).

#### 3.3.1 Spectral power

The mean frequency of cortical oscillations in PD patients decreases over the course of the disease. In a study involving PD patients at the earliest (drug‐naïve) disease stage, oscillatory slowing was already present, most pronounced over the posterior brain regions (Stoffers et al., 2007). When more advanced PD patients were studied, oscillatory slowing was hardly influenced by DRT (Stoffers, Bosboom, Wolters, Stam, & Berendse, 2008). Longitudinal analysis of PD patients revealed increases in band power of the “slower” frequencies (theta and alpha1 band), whereas band power of the “faster” frequencies (beta and gamma) decreased. The spectral slowing correlated with clinical progression of motor symptoms as well as global cognitive decline (Olde Dubbelink et al., 2013a). In a cross‐sectional analysis involving Parkinson's disease dementia (PDD) patients, spectral power had progressed toward diffuse slowing, independent of motor and cognitive scores (Bosboom et al., 2006; Ponsen, Stam, Bosboom, Berendse, & Hillebrand, 2012). The spectral slowing in PDD patients could at least partly be reversed by treatment with the cholinesterase inhibitor rivastigmine (Bosboom, Stoffers, Stam, Berendse, & Wolters, 2009). MEG‐derived spectral markers may help in predicting conversion to PDD: lower beta band power at baseline was the strongest predictor for conversion to PDD over a period of 7 years, followed by peak frequency and theta power. Moreover, the combination of impaired fronto‐executive task performance and low beta band power strongly increased the risk of conversion to PDD in this source‐space study (hazard ratio of 27.3 for both risk factors vs. none; Olde Dubbelink et al., 2014).

Stimulation of the STN can have widespread effects on oscillatory brain activity in multiple frequency bands. Whole‐brain average cortical frequency has been shown to increase upon stimulation of the STN (Cao et al., 2015). In sensors overlying the central sulcus, power in the beta band and of the mu rhythm decreased nonsignificantly, but the lowering in mu rhythm power (9–13 Hz in this study) correlated positively with relief of rigidity (Airaksinen et al., 2012). In another study, suppression of 9–13 Hz power (band width in line with (Airaksinen et al., 2012)) in posterior cortical brain regions and 8–30 Hz power in right temporal regions correlated positively with DBS‐related global motor improvement (Cao et al., 2015, 2017). In frontal and parietal regions, an increase in gamma band power has been reported following DBS, which in frontal regions correlated negatively with the improvement of total motor function (Cao et al., 2017)**.**

#### 3.3.2 Functional connectivity

In sensor‐space studies, local FC can be estimated by averaging FC values for all possible pairs of sensors within a given region of interest (ROI), whereas between‐ROI FC can be estimated by averaging FC for all possible pairs of sensors between ROIs. In a sensor‐space study, recently diagnosed (drug‐naïve) PD patients showed an overall higher local and between‐ROI alpha1 FC compared to controls (measured using synchronization likelihood (SL; (Stam & Van Dijk, 2002; Stoffers, Bosboom, Wolters, et al., 2008) an FC measure that captures both linear and nonlinear interactions). When moderately‐advanced PD patients were compared with controls, higher local functional connectivity (SL in one study and the phase lag index (PLI; less sensitive to volume conduction) in another) was found in PD patients, involving the theta, alpha1, alpha2, and beta band (Cao et al., 2018; Stoffers, Bosboom, Wolters, et al., 2008). Motor symptom severity and disease duration were positively associated with higher local and between‐ROI SL‐values (Stoffers et al., 2008). Furthermore, in one study DRT further increased between‐ROI beta band FC, as well as local FC in the range of 4–30 Hz in association with clinical motor improvement (especially over centroparietal brain regions; Stoffers, Bosboom, Wolters, et al., 2008). These findings are in contradiction with the findings of Cao and colleagues, who found the higher alpha PLI in PD patients to normalize upon DRT administration, in correlation with UPDRS‐III improvement (Cao et al., 2018). This discrepancy could perhaps be explained by a differential response to DRT observed by Stoffers and coworkers: in the majority of patients, already elevated levels of resting‐state local FC (4–30 Hz) further increased, but in patients with a strong improvement in motor function local beta band FC decreased (Stoffers, Bosboom, Wolters, et al., 2008). It was speculated that the differential response to DRT points at differences in the susceptibility to the development of response fluctuations and/or dyskinesias.

Longitudinal follow‐up of PD patients using the PLI in source‐space (the average PLI from a ROI with all other ROIs) revealed a higher baseline alpha1 PLI in cortical temporal regions in PD compared to controls. With disease progression, however, the initial changes in alpha1 PLI reversed, and an additional global decrease in alpha2 PLI appeared. These longitudinal changes correlated positively with worsening motor and global cognitive function. Interestingly, changes in alpha1 and alpha2 PLI in lower temporal regions, but not in the beta band, correlated with motor impairment (Olde Dubbelink et al., 2013b). Additional connectivity measures that have been used in source‐space analysis to demonstrate cross‐sectional differences between PD patients and controls include the phase locking value (PLV (Lachaux et al., 1999); comparable to PLI but sensitive to volume conduction/field spread) and directed Phase Transfer Entropy (dPTE; (Lobier, Siebenhuhner, Palva, & Palva, 2014)), a measure of directed connectivity. The PLV study demonstrated that during a working memory task, PD patients had significantly lower alpha band (9–16 Hz) PLV within the left‐hemispheric fronto‐temporal circuitry compared to controls, which correlated negatively with verbal working memory performance (Wiesman et al., 2016). The dPTE has been used to reveal lower beta band directed connectivity from posterior cortical brain regions toward frontal and subcortical brain regions in PD versus controls. In this study, lower directed connectivity from posterior cortical regions with the rest of the brain correlated with poor global cognitive performance in PD patients (Boon et al., 2017).

Comparison of a cohort of PDD patients with nondemented PD patients using two different processing pipelines led to conflicting outcomes that could at least partly be explained by differences in methodology (Bosboom, Stoffers, Wolters, Stam, & Berendse, 2009; Ponsen et al., 2012): in the first study, analysis was based on (ten) clusters of extracranial sensors and SL was used as FC measure. Compared to PD, PDD was characterized by lower fronto‐temporal SL in lower frequency bands (delta, theta and alpha1), and higher left‐sided parieto‐occipital SL in the higher frequency bands (alpha2 and beta; Bosboom, Stoffers, Wolters, et al., 2009). In the second (source‐level) analysis, FC was calculated using PLI. In the PDD group, PLI between pairs of regions was generally lower for the delta, alpha and beta band, and higher in the theta band. In the gamma band, differences went both ways (Ponsen et al., 2012).

#### 3.3.3 Topological organization

Olde Dubbelink et al. (2014) characterized the topological organization of PD brain networks in source‐space using graph analysis techniques. In early‐stage PD patients, lower local integration with preserved global efficiency of the whole‐brain network has been observed in the delta band. A longitudinal analysis demonstrated a tendency toward a more random brain topology, in which both local integration (multiple frequency bands) and global efficiency (alpha2 band) were affected. Worsening global cognition was associated with more random topology in the theta band, and motor dysfunction was associated with lower alpha2 global efficiency. In contrast to the more conventional application of graph analysis techniques, minimum spanning tree (MST) analysis is free of threshold and normalization biases. MST analysis revealed a progressive decentralization of the network configuration, starting in the early‐stage, untreated patients, which correlated with deteriorating motor function and cognitive performance (Olde Dubbelink, Hillebrand, Stoffers, et al., 2014).

## 4 DISCUSSION

In this review of the MEG literature on PD, we provide an overview of the neurophysiological characteristics of PD, their relationship with clinical motor and nonmotor symptoms, the effect of disease progression, and the influence of treatment on these characteristics. The design of the studies included in this review is very diverse, regarding both the MEG‐recordings itself (e.g., task‐based vs. resting‐state, eyes‐closed vs. eyes‐open, MEG signals alone or in relation to other measures, such as LFPs from the STN) and data analysis (e.g., source‐space vs. sensor‐space, different FC measures). Despite these challenging differences in data analytical approaches, we were able to extract several robust findings.

Motor‐network focused studies have uncovered a tremor network involving the motor cortex. In addition, these studies support the notion that, in contrast with the pathophysiology of bradykinesia and rigidity, not only basal‐ganglia‐cortical motor circuits, but also cerebello‐thalamo‐cortical circuits are important for PD‐related tremor (for further reading see (Helmich, 2018)). Another robust finding is the presence of functional loops between the STN and the temporal lobe (alpha band) and the STN and the sensorimotor cortex (beta and gamma band), although the clinical relevance and the effect of DRT on these loops remain to be established. Furthermore, as illustrated in Figures 2 and 3, the neurophysiological characteristics of the PD brain may vary over the course of the disease. For motor network‐focused studies this could be exemplified by increased cortical motor beta band power early in the disease and decreased cortical motor beta band power later in the disease. Whole‐brain studies showed a gradual slowing of the power spectrum and an initial increase in functional connectivity, which decreased over time in relation to disease progression, especially cognitive decline. Posterior cortical dysfunction seems to play a crucial role here (Boon et al., 2017; Olde Dubbelink, Hillebrand, Twisk, et al., 2014; Stoffers et al., 2007). Treatments such as DRT and rivastigmine generally normalized disrupted neurophysiological characteristics in both research fields, although many discrepancies exist, for example the increase in cortical motor beta power upon DRT (Heinrichs‐Graham, Kurz, et al., 2014), versus the decrease observed upon DBS (Abbasi et al., 2018; Luoma et al., 2018), or the differential effect of DRT on whole‐brain functional connectivity (Cao et al., 2018; Stoffers, Bosboom, Wolters, et al., 2008). Potential explanations for these discrepancies include methodological differences and differences in the underlying neurophysiological characteristics between PD patients (Figures 2 and 3).

When comparing the MEG findings discussed in this review with the EEG studies recently reviewed by Geraedts and colleagues (Geraedts et al., 2018), there is a prominent agreement on the link between spectral slowing and cognitive decline. Lower peak frequency and higher delta/theta power were the best predictors for future conversion to PDD in longitudinal EEG studies (Caviness et al., 2015; Cozac et al., 2016; Klassen et al., 2011; Latreille et al., 2016) and in an MEG study a lower beta band power was the best predictor (Olde Dubbelink, Hillebrand, Twisk, et al., 2014). The effect of DRT on whole‐brain power was inconclusive for both EEG (e.g., (Mostile et al., 2015) and MEG studies (Stoffers, Bosboom, Wolters, et al., 2008)), as well as the relationship between EEG/MEG‐findings and UPDRS‐III scores. Although EEG‐based longitudinal functional connectivity studies are missing, a few cross‐sectional studies hint at lower functional connectivity and network disruptions in cognitively disturbed PD patients (Hassan et al., 2017; Utianski et al., 2016), in accordance with the results of MEG studies (Olde Dubbelink et al., 2013b; Olde Dubbelink, Hillebrand, Stoffers, et al., 2014; Ponsen et al., 2012).

The results section of this review reflects the clear distinction between motor network‐focused MEG research and whole‐brain MEG research. Although this distinction often leaves little room for direct comparisons, both fields do share common grounds and we will further explore these in the next two sections.

### 4.1 Motor network‐focused research from a whole‐brain point of view

Beta band hypersynchrony within the STN and the basal ganglia‐thalamo‐cortical, cortico‐cortical and cerebro‐muscular loops is a well‐established electrophysiological phenomenon in PD, not only in the MEG field (Brown, 2003; Hammond, Bergman, & Brown, 2007; Kühn, Kupsch, Schneider, & Brown, 2006; Salenius et al., 2002; Stoffers, Bosboom, Deijen, et al., 2008). It has been suggested that the changes in beta band power/connectivity in PD might be a causal mechanism underlying the motor symptoms bradykinesia and rigidity, also considering the indirect evidence that treatment (either DRT or high‐frequency DBS) alleviates symptoms and at the same time causes a normalization of local band power and interregional coupling of beta activity (Hammond et al., 2007; Heinrichs‐Graham, Kurz, et al., 2014; Levy et al., 2002; Silberstein et al., 2005). However, there is no clear evidence that beta band synchronization directly accounts for the motor deficits in PD. Neurophysiological changes in motor network studies did not correlate with UPDRS‐III scores when recorded during the resting state (Abbasi et al., 2018; Litvak et al., 2011; Pollok et al., 2012; Pollok et al., 2013; Vardy et al., 2011). Furthermore, several unexpected negative correlations were observed when late‐stage PD patients were recorded during isometric contraction or a motor task of the forearm in the DRT‐OFF state (Hirschmann, Ozkurt, et al., 2013; Pollok et al., 2013). It has therefore been speculated that excessive beta band power and/or connectivity may not represent a pathological disinhibition with an anti‐kinetic effect, but could rather be interpreted as a compensatory mechanism that becomes redundant when DRT is administered (Hirschmann, Ozkurt, et al., 2013; Pollok et al., 2013). Hyperconnectivity has also been demonstrated in whole‐brain (both source‐space and sensor‐space) studies in the early stages of PD, most pronounced in the alpha1 band (Olde Dubbelink et al., 2013b; Stoffers, Bosboom, Deijen, et al., 2008). The interpretation of hyperconnectivity in early disease stages is not trivial and the discussion on this matter takes place in a broader context than that of PD only (de Haan, Mott, van Straaten, Scheltens, & Stam, 2012; Hillary & Grafman, 2017). Both pathological disinhibition and compensatory mechanisms may lead to higher FC values, but only a compensatory mechanism would be a purposeful reaction to a pathological process. However, it is unlikely that the latter mechanism is the sole explanation, since the majority of the studies in the present review did not show a positive correlation between higher FC and better clinical performance (Litvak et al., 2011; Pollok et al., 2012; Pollok et al., 2013; Stoffers, Bosboom, Deijen, et al., 2008; Vardy et al., 2011).

The functional subdivision between low and high‐beta frequencies might be of value in unraveling the relationship between interregional coupling of beta activity and clinical functioning. Whereas dopaminergic treatment mainly affected low‐beta spectral power in the STN, STN‐cortical coherence was strongest in the high‐beta band frequencies and was not modulated by levodopa (Litvak et al., 2011; van Wijk et al., 2016). Perhaps more complex functional interactions, such as cross‐frequency coupling (see also, (Tewarie et al., 2016)), could play a role in the pathophysiology of PD motor symptoms. Cross‐frequency coupling was previously found within the STN (van Wijk et al., 2016) and within the motor cortex ((de Hemptinne et al., 2013), but see also (Cole et al., 2017)) but not between these two structures.

Alternatively, negative correlations such as between M1‐STN beta band synchrony and UPDRS‐III scores could merely reflect normal physiology, in which case one would expect healthy individuals to show stronger M1‐STN coherence than PD patients (Hirschmann, Ozkurt, et al., 2013). Obviously, it is not possible to perform invasive recordings of brain activity in controls to confirm this, but a case study in an obsessive–compulsive disorder patient, treated with STN‐DBS, confirmed the presence of a high STN‐motor cortical connectivity in the beta band (Wojtecki et al., 2017). Furthermore, advances in source reconstruction techniques, such as beamforming, increasingly allow the study of subcortical regions by means of MEG (Boon et al., 2017; Hillebrand, Nissen, et al., 2016; Jha et al., 2017). At this point, however, additional methodological and experimental studies are necessary to evaluate the ability of beamformer techniques to reliably distinguish between individual subcortical brain regions.

Another important consideration is that the local neurophysiological processes observed in the motor network take place in a brain that is both structurally (Braak et al., 2003) and functionally (Olde Dubbelink et al., 2013a; Olde Dubbelink et al., 2013b) affected by PD on a whole‐brain scale. The interpretation of correlations between neurophysiological changes and motor symptoms is further complicated when studying the effect of DRT, since DRT can have varying effects on cortico‐cortical functional connectivity, dependent on disease stage and/or degree of UPDRS motor response to DRT (Stoffers, Bosboom, Deijen, et al., 2008).

Thus, neurophysiological changes on a whole‐brain scale may have directly or indirectly influenced findings in motor network‐focused MEG studies. Whole‐brain studies have demonstrated that neurophysiological changes associated with PD motor symptoms are not restricted to the “classical” motor network, which may have influenced findings *directly*: the slowing of beta band oscillations in the motor cortex observed in motor network‐focused studies in relatively advanced‐stage patients (Heinrichs‐Graham, Kurz, et al., 2014; Salenius et al., 2002) may in fact be part of the more general process of cortical oscillatory slowing (Olde Dubbelink et al., 2013a; Stoffers et al., 2007). Along the same line, the higher beta band functional connectivity between cortical motor regions (Heinrichs‐Graham, Kurz, et al., 2014) should be considered against the background of global increases in beta band cortico‐cortical FC that have been observed both using EEG and MEG in moderately advanced PD patients, and which correlated with both bradykinesia sub scores and disease duration (Silberstein et al., 2005; Stoffers, Bosboom, Deijen, et al., 2008). In contrast, in early disease stages larger beta band power has been observed in cortical motor regions in both PD patients and animal models of PD (Brazhnik et al., 2012; Degos, Deniau, Chavez, & Maurice, 2008; Hall et al., 2014; Javor‐Duray et al., 2015; Pollok et al., 2012), yet this has not been mirrored by the results of whole‐brain studies (Olde Dubbelink et al., 2013a; Stoffers et al., 2007).

Variability in ongoing brain activity contributes to the way the brain responds to certain sensory stimuli and therefore might *indirectly* influence differences in event‐related/induced motor responses between controls and PD patients (Sadaghiani, Hesselmann, Friston, & Kleinschmidt, 2010). Furthermore, whole‐brain band power changes are known to confound estimates of coherence between two neurophysiological signals and can thereby influence findings in motor network MEG studies (Schoffelen & Gross, 2009). In studies that estimated motor CMC, beta band power in cortical motor regions (and possibly also global beta band power) also differed between PD patients and controls and could therefore have impacted the CMC findings (Pollok et al., 2013; Salenius et al., 2002). In addition, the occipital dominant alpha band rhythm, mainly present when the eyes are closed, may dilute differences observed in the motor network studies (Luoma et al., 2018).

The interpretation of cortico‐subcortical interactions in DBS patients is hampered by the fact that these patients are generally in an advanced stage of disease and therefore have often received high doses of DRT for several years. Chronic DRT is known to influence cortical oscillations via neuronal plasticity (Degos et al., 2008). Furthermore, a longitudinal evaluation of the effect of STN‐DBS on beta band oscillations within the STN, coherence with cortical regions, and cortical oscillations along the disease course has not been performed yet. Therefore, when studying cortico‐subcortical coherence, the effects of the underlying disease, chronic use of medication and DBS itself on whole‐brain cortical oscillations should be taken into account.

### 4.2 Whole‐brain research: Toward a more focused approach

In whole‐brain MEG studies in PD, global oscillatory slowing, widespread changes in the strength of functional connectivity within and between brain areas, and a disruption of functional brain network organization have been observed. The consistent relationship between these findings and cognitive decline, motor dysfunction and disease duration support the notion that these whole‐brain neurophysiological changes may represent a general marker of the disease processes underlying PD (Bosboom et al., 2006; Olde Dubbelink et al., 2013a; Olde Dubbelink, Hillebrand, Stoffers, et al., 2014; Stoffers et al., 2007), a conclusion that is further supported by the results of EEG studies (Fonseca, Tedrus, Letro, & Bossoni, 2009; He et al., 2017; Morita, Kamei, Serizawa, & Mizutani, 2009). However, the mechanisms that lead to these widespread neurophysiological changes remain unknown, as well as the way in which these neurophysiological changes induce the clinical symptoms of PD, particularly the nonmotor symptoms.

There is increasing evidence to suggest that cortical neurophysiological changes in PD find their origin in subcortical brain regions. In early‐stage PD, involvement of brainstem dopaminergic, noradrenergic and serotonergic projection systems may be important factors that contribute to cortical oscillatory slowing (Bosboom et al., 2006; Bosboom, Stoffers, & Wolters, 2004). In later disease stages—including PD dementia—local cortical Lewy body and tau pathology, local pathology in thalamo‐cortical circuits (Freunberger, Werkle‐Bergner, Griesmayr, Lindenberger, & Klimesch, 2011; Steriade, Gloor, Llinas, Da Silva, & Mesulam, 1990), and degeneration of the cholinergic nucleus of Meynert (Bosboom, Stoffers, Stam, et al., 2009; Hepp et al., 2017) may contribute to cortical neurophysiological changes in PD.

Observations that highlight the importance of cortico‐subcortical interactions in PD include the influence of STN‐DBS on whole‐brain oscillations (Airaksinen et al., 2012; Cao et al., 2015; Cao et al., 2017)**,** the possible influence of STN‐DBS on a multitude of nonmotor symptoms (Castrioto, Lhommée, Moro, & Krack, 2014) and the presence of an STN‐temporal network in the alpha band that shows PD‐related functional changes and is influenced by movement (Hirschmann et al., 2011; Litvak et al., 2011; Olde Dubbelink, Hillebrand, Twisk, et al., 2014; Oswal et al., 2013a; Oswal, Jha, et al., 2016). Future whole‐brain studies could build on these observations by including estimation of cortico‐subcortical interactions using source reconstruction techniques, and correlate findings to both motor and nonmotor symptoms.

The neurophysiological changes observed in whole‐brain resting‐state studies correlated with both motor and nonmotor symptoms of PD (Bosboom et al., 2006; Olde Dubbelink et al., 2013a; Olde Dubbelink et al., 2013b; Olde Dubbelink, Hillebrand, Stoffers, et al., 2014; Stoffers, Bosboom, Deijen, et al., 2008), hence the interpretation of these changes might be more ambiguous than the observations in task‐related conditions. On the other hand, whole‐brain resting‐state neurophysiological changes might be a more accurate marker of the underlying disease process. A reliable (noninvasive) in vivo marker of the disease process can be used to predict the disease course in individual patients and to monitor the effects of modulatory techniques such as DBS or future disease‐modifying drugs.

The approach of focusing on average FC from a ROI with all other regions in a whole‐brain analysis might be too diffuse to pick up changes restricted to certain sub systems. When trying to bridge the gap between the underlying disease and specific PD‐related symptoms—referred to as pathophysiology in this context—a more focused approach would be preferable. A seed‐based analysis could be used to confirm hypotheses that have arisen based on whole‐brain research. In addition, particular symptoms such as cognitive dysfunction in specific domains may be correlated to changes in (dynamic) connectivity between specific subnetworks (Kucyi, Hove, Esterman, Hutchison, & Valera, 2016; Park, Friston, Pae, Park, & Razi, 2017). A more focused approach can provide important additional information on the pathophysiology of specific disease‐related symptoms, which may prove useful for the development of symptomatic treatments, for example, targeting key brain regions or subnetworks using TMS or DBS. These exciting therapeutic possibilities are already being tested in PD patients (Freund et al., 2009; Manenti et al., 2016).

### 4.3 Clinical utility of MEG in PD

Of the robust findings we have presented in this review, up to now only MEG‐derived spectral markers (markers of spectral slowing) as predictors for conversion to PDD have potential for routine clinical use (Olde Dubbelink, Hillebrand, Twisk, et al., 2014). As these in‐vivo biomarkers of disease progression can also be derived from cheaper and more widely available EEG recordings (Geraedts et al., 2018), the need to include MEG in standard clinical care is currently low. However, with MEG, patients would benefit from a more comfortable and faster recording technique. In addition, when the higher spatial resolution of MEG over EEG is exploited, application of MEG in routine clinical care could become more rational (see (Hillebrand, Gaetz, Furlong, Gouw, & Stam, 2018) for further reading on the clinical application of MEG). Future studies are required to establish whether measures of functional connectivity or brain network structure, which could be determined more reliably using MEG, can surpass spectral slowing as an in‐vivo biomarker of cognitive decline or disease progression in a broader sense.

The optimization of stimulation settings after DBS‐placement could also benefit from MEG‐recordings, both for nonmotor and motor effects. Potentially, beta band power in the sensorimotor cortex could serve as a biomarker for optimal motor effects, although the link between cortical beta oscillations and motor function is not clear yet (Abbasi et al., 2018; Luoma et al., 2018). Alternatively, a more dispersed cortical fingerprint could serve as a biomarker for optimal clinical (both motor and nonmotor) effects.

### 4.4 Conclusion

Macro‐scale neurophysiological changes in the PD brain have classically been studied from two different perspectives. Some research groups have studied PD‐related changes in the brain as a whole, while others have explored relationships between more localized brain activity and motor symptoms, thereby focusing on pathophysiological mechanisms. However, the two research fields are certainly not mutually exclusive and the knowledge gained from both approaches may even be complementary: motor network function is influenced by whole‐brain changes in neuronal activity related to the ongoing disease processes, whereas whole‐brain analysis may not fully capture local pathophysiological mechanisms underlying specific symptoms. Up to now, results of MEG studies have been very diverse and the application of MEG in standard clinical care is limited. Future studies that combine the merits of both approaches could increase reproducibility and interpretation of results, which will undoubtedly lead to valuable insights into the neuronal mechanisms underlying PD as well as into the pathophysiology of the broad range of clinical symptoms that characterize this disease.

## DISCLOSURES

Drs. Boon reports no disclosures. Drs. Geraedts received grant support from the Stichting Alkemade Keuls and Stichting ParkinsonFonds. Dr. ir. Hillebrand reports no disclosures. Dr. Tannemaat has been a member of scientific advisory board of Araim Pharmaceuticals and has stock options in the same company. Dr. Contarino has been a member of scientific advisory boards of Medtronic and Boston Scientific companies and has been an independent consultant for Medtronic for research and educational issues. The DBS center of the Haga Teaching Hospital/LUMC received compensation for DBS training activities from Medtronic and an unrestricted educational grant from Medtronic. Prof. Dr. Stam reports no disclosures. Prof. Dr. Berendse reports no disclosures.

## Supporting information


**Appendix S1**: Supporting information.
