# Supplementary material for: A systematic review of MEG‐based studies in Parkinson's disease: The motor system and beyond
Source: Hum Brain Mapp. 2019 Mar 7;40(9):2827–48. doi: 10.1002/hbm.24562 (PMC6594068; doi:10.1002/hbm.24562)
Supplement: Supplementary file 1 — Appendix S1: Supporting information. [file HBM-40-2827-s001.docx]

**Supplement A**

***PubMed***

(**("Parkinson Disease"[Mesh] OR "Parkinson Disease, Secondary"[Mesh:NoExp] OR "Parkinsonian Disorders"[Mesh:noexp] OR "parkinsonian"[tw] OR parkinsonian*[tw] OR "parkinsonism"[tw] OR parkinsonism*[tw] OR "parkinson disease"[tw] OR "parkinson's disease"[tw] OR "parkinsons disease"[tw] OR "Paralysis Agitans"[tw] OR parkinson*[tw]) AND ("Magnetoencephalography"[Mesh] OR "magnetoencephalography"[tw] OR magnetoencephalogr*[tw] OR "magneto-encephalography"[tw] OR magneto-encephalogr*[tw] OR "MEG"[tw]))**

***Embase***

(**(exp "Parkinson Disease"/ OR "Parkinsonism"/ OR "parkinsonian".mp OR parkinsonian*.mp OR "parkinsonism".mp OR parkinsonism*.mp OR "parkinson disease".mp OR "parkinson's disease".mp OR "parkinsons disease".mp OR "Paralysis Agitans".mp OR parkinson*.mp) AND ("Magnetoencephalography"/ OR "magnetoencephalography".mp OR magnetoencephalogr*.mp OR "magneto-encephalography".mp OR magneto-encephalogr*.mp OR "MEG".mp)) NOT conference review.pt**

***Web of Science***

(TS=**("Parkinson Disease" OR "Parkinsonism" OR "parkinsonian" OR parkinsonian* OR "parkinsonism" OR parkinsonism* OR "parkinson disease" OR "parkinson's disease" OR "parkinsons disease" OR "Paralysis Agitans" OR parkinson*) AND TI=("Magnetoencephalography" OR "magnetoencephalography" OR magnetoencephalogr* OR "magneto-encephalography" OR "magneto-encephalogr*" OR "MEG")) OR (**TI=**("Parkinson Disease" OR "Parkinsonism" OR "parkinsonian" OR parkinsonian* OR "parkinsonism" OR parkinsonism* OR "parkinson disease" OR "parkinson's disease" OR "parkinsons disease" OR "Paralysis Agitans" OR parkinson*) AND TS=("Magnetoencephalography" OR "magnetoencephalography" OR magnetoencephalogr* OR "magneto-encephalography" OR "magneto-encephalogr*" OR "MEG"))**

***Cochrane***

(**("Parkinson Disease" OR "Parkinsonism" OR "parkinsonian" OR parkinsonian* OR "parkinsonism" OR parkinsonism* OR "parkinson disease" OR "parkinson's disease" OR "parkinsons disease" OR "Paralysis Agitans" OR parkinson*) AND ("Magnetoencephalography" OR "magnetoencephalography" OR magnetoencephalogr* OR "magneto-encephalography" OR "magneto-encephalogr*" OR "MEG"))**

***Emcare***

(**(exp "Parkinson Disease"/ OR "Parkinsonism"/ OR "parkinsonian".mp OR parkinsonian*.mp OR "parkinsonism".mp OR parkinsonism*.mp OR "parkinson disease".mp OR "parkinson's disease".mp OR "parkinsons disease".mp OR "Paralysis Agitans".mp OR parkinson*.mp) AND ("Magnetoencephalography"/ OR "magnetoencephalography".mp OR magnetoencephalogr*.mp OR "magneto-encephalography".mp OR magneto-encephalogr*.mp OR "MEG".mp)) NOT conference review.pt**

***Academic Search Premier***

(**("Parkinson Disease" OR "Parkinsonism" OR "parkinsonian" OR parkinsonian* OR "parkinsonism" OR parkinsonism* OR "parkinson disease" OR "parkinson's disease" OR "parkinsons disease" OR "Paralysis Agitans" OR parkinson*) AND ("Magnetoencephalography" OR "magnetoencephalography" OR magnetoencephalogr* OR "magneto-encephalography" OR "magneto-encephalogr*" OR "MEG"))**

***ScienceDirect***

TITLE-ABSTR-KEY(**("Parkinson Disease" OR "Parkinsonism" OR "parkinsonian" OR parkinsonian* OR "parkinsonism" OR parkinsonism* OR "parkinson disease" OR "parkinson's disease" OR "parkinsons disease" OR "Paralysis Agitans" OR parkinson*) AND ("Magnetoencephalography" OR "magnetoencephalography" OR magnetoencephalogr* OR "magneto-encephalography" OR "magneto-encephalogr*" OR "MEG")**)

**Supplement B**

JBI Critical Appraisal Checklist for Case Series

Reviewer**……………………………………………………** Date**…………………………………………..**

Author**……………………………………………………….** Year**…………………………………………..** Record number**………**

Yes No Unclear Not

Applicable

1. Were there clear criteria for inclusion in the

case series?

1. Was the condition measured in a standard, reliable

way for all participants included in the case series?

1. Were valid methods for identification of the

condition for all participants included in the case

series?

1. Did the case series have consecutive inclusion of

participants?

1. Did the case series have complete inclusion of

participants?

1. Was there clear reporting of the demographics of

the participants in the study?

1. Was there clear reporting of clinical information

of the participants?

1. Were the outcomes or follow up results of cases

clearly reported?

1. Was there clear reporting of the presenting site(s)/

clinic(s) demographic information?

1. Was statistical analysis appropriate?
2. Was there clear reporting of MEG data acquisition

and analysis?*

Overall appraisal: Include Exclude Seek further info

**Minimum requirements: 1x ‘yes’ question 1-3, 2x ‘yes’ question 4-8, 1x ‘yes’ question 11.**

Comments (Including reason for exclusion)

­­­­­­­__________________________________________________________________________________

­­­­­­­__________________________________________________________________________________

** Minimum requirements: details MEG acquisition (system and sensor type, sampling frequency, position participant, description task/resting state), amount of data gathered (length and number of data segments), in case of source reconstruction: details on the atlas or grid/resolution used*

**Supplement C**

| **Author (year)** | **Title** | **Reason exclusion** | **JBI scores** |
| --- | --- | --- | --- |
| Anninos et al. (2000) | *Nonlinear analysis of brain activity in magnetic influenced Parkinson patients* | -No quantification of an MEG outcome parameter  -No statistics performed  -No reproducible MEG acquisition (item 11)  -Total JBI score too low, too low score for items 4-8 | Total: 3  Item 1-3: 1  Item 4-8: 1  Item 9-10: 1  Item 11: 0 |
| Park et al. (2009) | *Cortico-muscular coherence increases with tremor improvement after deep brain stimulation in Parkinson’s disease* | -No reproducible MEG acquisition (item 11)  -Total JBI too low | Total: 4  Item 1-3: 1  Item 4-8: 2  Item 9-10: 1  Item 11: 0 |
| Bock et al. (2013) | *Validity of subthalamic-cortical coherency observed in patients with Parkinson’s disease* | -Main goal of article was validation of method: Total -Total JBI score too low, too low score for items 4-8. | Total: 3  Item 1-3: 1  Item 4-8: 1  Item 9-10: 0  Item 11: 1 |
